# Protein–lipid Association in Lizard Chemical Signals

**DOI:** 10.1093/iob/obad016

**Published:** 2023-05-08

**Authors:** M Mangiacotti, S Baeckens, M Fumagalli, J Martín, S Scali, R Sacchi

**Affiliations:** Department of Earth and Environmental Sciences, University of Pavia, Via Taramelli 24, 27100 Pavia, Italy; Functional Morphology Lab, Department of Biology, University of Antwerp, Universiteitsplein 1, 2610 Wilrijk, Belgium; Evolution and Optics of Nanostructures Group, Department of Biology, Ghent University, 9000 Gent, Belgium; Department of Biology and Biotechnologies “L. Spallanzani”, University of Pavia, Via Ferrata 9, 27100 Pavia, Italy; Departamento de Ecología Evolutiva, Museo Nacional de Ciencias Naturales, CSIC, José Gutiérrez Abascal 2, E-28006 Madrid, Spain; Sezione Erpetologia, Museo di Storia Naturale di Milano, Corso Venezia 55, IT-20121 Milano, Italy; Department of Earth and Environmental Sciences, University of Pavia, Via Taramelli 24, 27100 Pavia, Italy

## Abstract

Chemical communication in terrestrial vertebrates is often built on complex blends, where semiochemical and structural compounds may form an integrated functional unit. In lizards, many species have specialized epidermal glands whose secretions are waxy, homogeneous blends of lipids and proteins, both active in communication. The intimate co-occurrence of such compounds allows us to hypothesize that they should undergo a certain degree of covariation, considering both their semiochemical role and the support-to-lipid function hypothesized for the protein fraction. In order to assess the occurrence and level of protein–lipid covariation, we compared the composition and complexity of the two fractions in the femoral gland secretions of 36 lizard species, combining phylogenetically-informed analysis with tandem mass spectrometry. We found the composition and complexity of the two fractions to be strongly correlated. The composition of the protein fraction was mostly influenced by the relative proportion of cholestanol, provitamin D_3_, stigmasterol, and tocopherol, while the complexity of the protein pattern increased with that of lipids. Additionally, two identified proteins (carbonic anhydrase and protein disulfide isomerase) increased their concentration as provitamin D_3_ became more abundant. Although our approach does not allow us to decrypt the functional relations between the proteinaceous and lipid components, nor under the semiochemical or structural hypothesis, the finding that the proteins involved in this association were enzymes opens up to new perspectives about protein role: They may confer dynamic properties to the blend, making it able to compensate predictable variation of the environmental conditions. This may expand the view about proteins in the support-to-lipid hypothesis, from being a passive and inert component of the secretions to become an active and dynamic one, thus providing cues for future research.

## Abstract (es)

(Italian) Riassunto

La comunicazione chimica nei vertebrati terrestri è spesso costituita da miscele complesse, in cui composti semiochimici e strutturali possono formare un“unità funzionale integrata. Nelle lucertole, molte specie hanno ghiandole epidermiche specializzate le cui secrezioni sono miscele cerose e omogenee di lipidi e proteine, entrambe attive nella comunicazione. L”intima coesistenza di tali composti lascia ipotizzare che essi debbano subire un certo grado di co-variazione, sia considerando il loro ruolo semiochimico, sia la funzione di supporto ai lipidi ipotizzata per la frazione proteica. Per valutare la presenza e il livello di covariazione proteine-lipidi, abbiamo confrontato la composizione e la complessità delle due frazioni nelle secrezioni della ghiandola femorale di 36 specie di lucertole, combinando l“analisi filogenetica comparativa con la spettrometria di massa. Abbiamo riscontrato una forte correlazione tra la composizione e la complessità delle due frazioni. La composizione della frazione proteica è stata influenzata soprattutto dalla proporzione relativa di colestanolo, provitamina D_3_, stigmasterolo e tocoferolo, mentre la complessità del pattern proteico è aumentata con quella dei lipidi. Inoltre, due proteine identificate (anidrasi carbonica e disolfuro isomerasi) hanno aumentato la loro concentrazione al crescere dell'abbondanza della provitamina D_3_. Sebbene il nostro approccio non consenta di decifrare le relazioni funzionali tra le componenti proteiche e lipidiche, né secondo l”ipotesi semiochimica né secondo quella strutturale, la scoperta che le proteine coinvolte in questa associazione sono enzimi apre a nuove prospettive sul ruolo delle proteine stesse: esse potrebbero conferire proprietà dinamiche alla miscela, rendendola capace di compensare le prevedibili variazioni delle condizioni ambientali. Questo può ampliare la visione delle proteine nell'ipotesi che esse siano di supporto ai lipidi, da componente passiva e inerte delle secrezioni a componente attiva e dinamica, fornendo così spunti per ricerche future.

## Abstract (de)

(Spanish) Resumen

La comunicación química en los vertebrados terrestres se basa a menudo en mezclas complejas, donde los compuestos semioquímicos y estructurales pueden formar una unidad funcional integrada. En los lagartos y lagartijas, muchas especies tienen glándulas epidérmicas especializadas cuyas secreciones son mezclas cerosas y homogéneas de lípidos y proteínas, siendo ambos compuestos activos en la comunicación. La co-ocurrencia estrecha de tales compuestos permite plantear la hipótesis de que debería existir un cierto grado de covariación, considerando los posibles papeles de la fracción proteica tanto como semioquímicos y como apoyo a la función de los lípidos. Con el fin de evaluar la ocurrencia y el nivel de covariación entre proteínas y lípidos, comparamos la composición y complejidad de estas dos fracciones en las secreciones de la glándula femoral de 36 especies de lagartos y lagartijas, combinando análisis filogenéticos con espectrometría de masas en tándem. Encontramos que la composición y la complejidad de las dos fracciones estaban fuertemente correlacionadas. La composición de la fracción proteica estuvo influenciada principalmente por la proporción relativa de colestanol, provitamina D_3_, estigmasterol y tocoferol, mientras que la complejidad del patrón proteico aumentó con el de los lípidos. Además, dos proteínas identificadas (anhidrasa carbónica y proteína disulfuro isomerasa) aumentaban su concentración a medida que la provitamina D_3_ era más abundante. Aunque nuestro enfoque no permite descifrar las relaciones funcionales entre los componentes proteináceos y lipídicos, ni bajo la hipótesis semioquímica, ni bajo la estructural, el hallazgo de que las proteínas involucradas en esta asociación serían enzimas abre nuevas perspectivas sobre el papel de las proteínas. Estas podrían conferir propiedades dinámicas a la mezcla, haciéndola capaz de compensar la variación esperada debida a las condiciones ambientales. Estos resultados pueden ampliar la visión del papel de las proteínas en la hipótesis de apoyo a los lípidos, pasando de ser un componente pasivo e inerte de las secreciones a convertirse en uno activo y dinámico, proporcionando así ideas para investigaciones futuras.

## Introduction

The chemical signals of animals often consist of a complex mixture of different classes of molecules with distinct properties (Alberts 1992; Lei and Vickers 2008; Wyatt 2010; Clifford and Riffell 2013; Junker et al. 2018). Such chemical blends typically combine volatile compounds, which spread easily and may travel far, with more heavy and stable non-volatiles, which may serve as the complementary or additional part of the information conveyed by the volatile fraction, or may favor signal stability, and prolong the durability of the volatile counterparts, or both (Alberts 1992; Nehring et al. 2013; Mucignat-Caretta and Caretta 2014; Wyatt 2014).

From an adaptive perspective, chemical mixtures, being built from different molecules, can be considered a multivariate trait (Lande 1979; Lande and Arnold 1983; Blows 2007; Murren 2012). If so, and blends represent a single, integrated functional unit, then selective forces are likely not acting separately on each single component, but rather on the covariation matrix of the mixture (Murren 2012; Armbruster et al. 2014; Felice et al. 2018). Indeed, several studies have shown that different components of a chemical mixture may influence each other (e.g., Briand et al. 2004; Mucignat-Caretta and Caretta 2014; Romero-Diaz et al. 2021), or that key messages (i.e., information) may be encoded in the relative proportions of compounds (e.g., Ozaki et al. 2005; di Mauro et al. 2015). These considerations highlight the need of an integrated approach in the evolutionary study of chemical blends used in inter- and intra-specific communication (Junker et al. 2018).

Over the last 30 years or so, lizards have become a model species to investigate the evolutionary forces shaping vertebrate chemical communication (e.g., Alberts 1991; Alberts et al. 1993; Martín and López 2000; Pruett et al. 2016; Baeckens et al. 2017; García-Roa et al. 2017a, 2017b; Campos et al. 2020; Ruiz–Monachesi et al. 2020; Baeckens and Whiting 2021; Mangiacotti et al. 2021). The reason for its popularity is partly due to the pivotal role of the chemical sensory modality in this taxon, which is not only characterized by readily observable chemoreception behavior of lizards (i.e., tongue-flicking; Schwenk 1995; Baeckens et al. 2017), but also by the occurrence, in most species, of epidermal glands in the cloacal region (García-Roa et al. 2017a; Cole 1966), used for inter- and intra-specific signaling (Martín and López 2014; Mayerl et al. 2015). Among them, femoral glands occur in the lizard lineages which do not suffer limb reduction (García-Roa et al. 2017a) and has been widely studied (Mayerl et al. 2015). Femoral glands consist of two symmetric series of structures along the inner part of the thighs (Cole 1966), which produce a waxy mixture of proteins (low-volatile) and lipids (high-volatile), in variable proportions (Alberts 1990; Escobar et al. 2001; Mangiacotti et al. 2019b). Both components are functional in communication (Alberts and Werner 1993a; Martín and López 2014, 2015; Mangiacotti et al. 2019a, 2020). The lipid fraction has been the focus of most studies (e.g., Alberts et al. 1992; Khannoon et al. 2011; Heathcote et al. 2014; Martín et al. 2015a; Pruett et al. 2016; Zozaya et al. 2019; Donihue et al. 2020), which resulted in a relatively good understanding of the identity and partly of the signaling role of the involved lipids (Weldon et al. 2008; Martín and López 2011, 2014, 2015). Aside from specific molecules, many studies have also analyzed the variability of the whole lipid blend, and its association with environmental conditions (Martín et al. 2015b; Martín et al. 2017; Baeckens et al. 2018a; Ortega et al. 2019; Campos et al. 2020), phylogeny (Baeckens et al. 2018, 2018a), or individual traits (López and Martín 2005; Labra 2006; Ortega et al. 2019).

Conversely, proteins from femoral glands secretions have been far less studied (Martín and López 2014; Mayerl et al. 2015; Mangiacotti et al. 2017; Romero-Diaz et al. 2020), although they may represent most of the secretion mass (Alberts 1990; Mangiacotti et al. 2019b), they are homogeneously mixed with lipid blend (Alberts 1990; Mangiacotti et al. 2017), and can be detected by lizards (Alberts and Werner 1993a; Cooper et al. 2002; ). On this basis, two main, not exclusive, functions have been theorized for secretion proteins: On the one hand, the support-to-lipid hypothesis, which states that proteins represent an inert structural matrix that holds and protects lipids in the secretion from fading (Cole 1966); and on the other hand, the semiochemical hypothesis, which considers the proteinaceous fraction to be directly involved in signaling (Alberts 1990; Alberts and Werner 1993a). Indeed, behavioral studies on the green iguana (*Iguana iguana*; Alberts and Werner 1993a), the common wall lizard (*Podarcis muralis*; Mangiacotti et al. 2019a), and Cyren's Rock Lizard (*Iberolacerta cyreni*; Mangiacotti et al. 2020) showed that proteins could play an active role in lizard chemical communication, at least conveying identity-related information. Recently, using mass-spectrometry coupled with database-searching, proteins from femoral glands have been preliminary characterized in the marine iguana, *Amblyrhynchus cristatus* (Tellkamp et al. 2020), and the sand lizard, *Lacerta agilis* (Ibáñez et al. 2022). Although both studies failed in finding proteins immediately relatable to semiochemical functions, they underscored their role in supporting the lipophilic fraction, with potential binding, regulatory, anti-oxidant, antibacterial, and immune functions (Tellkamp et al. 2020; Ibáñez et al. 2022).

Overall, the above findings suggest the idea that lipids and proteins may show a certain degree of covariation. Unfortunately, no study has investigated whether and how the composition of the lipophilic fraction of lizard chemical signals is correlated to that of its proteinaceous fraction. By systematically focusing on solely one aspect of the lipid–protein mix, our comprehension of lizard chemical communication has been potentially biased, preventing us from investigating the potentiality for covariation effects. In the present study we examined, for the first time, the pattern of covariation between lipids and proteins from femoral glands secretions, with the specific aim of assessing whether the composition of the proteinaceous fraction reflects the variability of the lipophilic counterpart. We focused on the Lacertidae family, an Old World lizard group distributed in Europe, Africa, and Asia, including about 350 species of small-to-medium-sized lizards (with few exceptions) (Sindaco and Jeremcenko 2008; Roll et al. 2017; Garcia-Porta et al. 2019). Chemical communication plays an important role in this group (Baeckens et al. 2015; Mangiacotti et al. 2021), and it has been extensively studied (Baeckens 2019). We assembled a comprehensive dataset of the protein and lipid composition of femoral gland secretions of 36 lacertid lizard species, and analyzed the protein–lipid relation under a phylogenetic comparative framework. A tentative identification of the proteins targeted by the comparative analysis was carried out using mass-spectrometry coupled with database-searching.

## Material and methods

### Dataset assembly

In order to compare the covariation in lipid and protein components of lizard femoral gland secretions, we set the observation scale to the species level, and combined already published data to new, *ad hoc* ones, which covered information gaps.

Overall, the dataset included complete information about composition and richness of both proteins and lipids from femoral gland secretions of 36 lacertid species (Table S1; note on sample collections are summarized in supplementary materials), together with a recent reconstruction of their phylogenetic relations (Garcia-Porta et al. 2019). Protein and lipid samples came from the same individuals (only males) and populations (Baeckens et al. 2018a; Mangiacotti et al. 2021). The final species list was mainly driven by data availability, mostly concerning the protein fraction, which was the actual limiting factor. Below the detailed description of the data collected.

#### Proteins

Information about femoral gland proteins were obtained from already published data (Mangiacotti et al. 2021), and consisted in species-level one-dimensional normalized average electrophoretic profiles (electrophoretogram, EPG) from 36 lacertid species. Each specific EPG was made of 300 values representing the relative amount of protein clusters ordered by their molecular weight (Garfin 2009). Since protein identification is still challenging because it requires species-specific databases to enable the interpretation of tandem mass spectrometry data (Eng et al. 2011; Tellkamp et al. 2020), as a working hypothesis, we assumed EPG to represent a raw proxy of the protein composition (as in Alberts 1991; Alberts et al. 1993; Mangiacotti et al. 2019b, 2021). From EPG, we also obtained an estimation of the complexity of the protein mix as (1) the number of identifiable peaks (R_P_; Table S1), corresponding to the number of bands in the electrophoretic profile, and therefore correlated to protein richness, and (2) the Shannon's entropy (H_P_; Table S1), an information measure of EPG complexity. R_P_ was extracted from each EPG using the function detect_localmaxima of the R package scorepeak (Ochi 2019), setting the searching window to three (*w* = 3) and selecting as true peaks those being at least 10% as high as the highest peak. H_P_ was directly computed from EPG using the formula ${H}_P = \mathop \sum \nolimits_i {p}_i \cdot {\rm{log}}( {{p}_i} )$, where *p_i_* represents the normalized protein abundance for the *i*th molecular weight bin (all EPGs were series of 300 values; Mangiacotti et al. 2021). All operations were implemented in R v3.5.2 (R Core Team 2018) adapting the functions available in Mangiacotti et al. (2019b).

#### Lipids

For each of the 36 lacertid species for which protein data were available, we retrieved three kinds of information about lipids. Firstly, we obtained average specific lipid composition at chemical class level (Weldon et al. 2008) from already published data (Baeckens et al. 2018b). For each species, we organized all compounds in 11 main classes, based on their chemical nature and the length of their carbon chains (i.e., volatility; Baeckens et al. 2018a): low weight alcohols (C ≤ 16), high weight alcohols (C > 16), aldehydes, low weight fatty acids (C ≤ 16), high weight fatty acids (C > 16), waxy esters, furanones, ketones, terpenoids, tocopherols, and steroids. This grouping allowed, including both identified and confirmed compounds, and compounds that, although their specific identities were unknown, could be assigned to a chemical class based on their mass spectra characteristics. Moreover, with this grouping, we characterized the lipid fraction at the same resolution already used in previous studies (Gabirot et al. 2008; Khannoon et al. 2011, 2013; Martín et al. 2013, 2015b; Baeckens et al. 2017; García-Roa et al. 2017b; Baeckens et al. 2018, 2018a). The reported average percentage in each class was used as the lipid profile of each species. Secondly, we focused on the relative abundance of eight main compounds which are known to be active in chemical communication or in holding signal efficacy (Weldon et al. 2008; Martín and López 2014, 2015; García-Roa et al. 2017b): cholesterol, campesterol, stigmasterol, ergosterol (provitamin D_2_), 9,12-octadecadienoic acid (linoleic acid), α-tocopherol (vitamin E), cholestanol, and cholesta-5,7-dien-3-ol (provitamin D_3_). Despite absent in some species, the chosen compounds are shared (at different concentration) among all individuals of the same species (Martín and López 2014; García-Roa et al. 2017b), not leading to artifact average profiles. For half of the species, data were directly available from García-Roa et al. (2017b). For the remnant species, for which data were not available from literature, the original GC-MS data from Baeckens et al. (2018a) were re-analyzed, and the relative abundance of the targeted compounds was calculated as the relative proportion of the total ion current, in exactly the same way as in García-Roa et al. (2017b). Thirdly, we included two overall measures of the complexity of the lipid mixture (Baeckens et al. 2018a): the total number of distinct compounds (identified and unidentified) found in the GC-MS analysis defined the lipid richness R_L_ (Baeckens et al. 2018a); the Shannon's entropy of the lipid profiles (H_L_), computed applying the same formula used for H_P_, defined lipid complexity.

#### Phylogeny

The phylogenetic tree, including all the studied species was obtained starting from the phylogeny of lacertid lizards available in Garcia-Porta et al. (2019). The original tree, based on phylogenomic analysis of 246 species, was pruned to fit the 36 species of the present study using the keep.tip function of the ape R package (Paradis and Schliep 2019).

### Statistical analysis

Before analysis, some data were transformed to account for their compositional nature (Aitchison 1982; van den Boogaart and Tolosana-Delgado 2013). Notably, we applied the centred-log-ratio transformation (Aitchison 1982) to the matrix of normalized EPG (proxy for protein composition) and that of lipid composition. Similarly, the matrix of the abundance of the eight main lipids was first closed (i.e., normalized to sum to one), in order to obtain the relative proportion of each compound compared to the others; then, the centred-log-ratio transformation was applied, obtaining the final matrix. In the above cases, a back-transformation of the data was applied when interpreting results (Aitchison 1982; van den Boogaart and Tolosana-Delgado 2013). R_P_, H_P_, R_L_, and H_L_ were kept in their original scale.

As a first step, we assessed the degree of covariation between proteins and lipids using a phylogenetic partial least square analysis (Adams and Felice 2014), implemented by the function phylo.integration of the R package geomorph (Adams and Otárola-Castillo 2013). This analysis quantifies the degree of covariation of two phenotypic trait matrixes, after accounting for the phylogenetic dependence among the observations (species), which is used to transform the original trait values under a Brownian motion model of evolution (Adams and Felice 2014). Besides being conventionally used as a “null-model” for trait evolution (Felsenstein 1985), Brownian motion was selected for analogy with the evolutionary mode of lipids from femoral gland secretions, as assessed at single compound level across the lizard tree (García-Roa et al. 2017b). The partial least squares output is a correlation coefficient (r_PLS_), whose statistical significance is assessed via permutations (Adams and Felice 2014). The approach is well suited for high-dimensional matrixes (i.e., more variables than observations; Adams and Felice 2014; Collyer et al. 2015), as it is the case for EPG (36 rows × 300 columns). We set the number of permutations to 9999. A second analysis was run considering EPG and the relative abundance of the main eight lipid compounds as covariating matrixes.

Secondly, we used a phylogenetic regression via generalized least squares (Adams 2014) to investigate whether the abundance of the eight specific lipids affects the EPG, and which molecular weight regions are more influenced. We used the function procD.pgls from geomorph package, with normalized EPGs as the response matrix and the absolute abundances of the eight main compounds as predictors. Significance of predictors was assessed via residual permutations, using marginal (type III) sum of square and cross product computation (Adams and Collyer 2015). Model predictions were then used to identify the EPG regions with the highest response to increase abundance of significant lipids.

Thirdly, phylogenetic regression was also used to correlate protein- to lipid-complexity: in turn, protein richness (R_P_) or complexity (H_P_) entered the model as dependent variables, with corresponding lipid richness (R_L_) and complexity (H_L_) used as the only predictor. Although a parametric model is available for univariate response variables, we preferred using the same, multivariate, distance-based analysis, to keep a homogeneous approach to parameter estimation and significance assessment, considering that the two methods give numerically identical results for univariate data (Adams 2014).

### Protein identification

As phylogenetic regression indicated a statistically significant relationship between EPG and the abundance of provitamin D_3_ (see results), we investigated the proteinaceous fraction in more detail with the aim to identify the specific proteins involved. First, we localized the molecular weight regions of EPG predicted to be related to provitamin D_3_ increase; then, we searched specific EPGs bearing the highest expression in those three weight regions. We found the EPG of *Podarcis muralis* to have such characteristics. Thus, we retrieved the original protein samples used in the analysis from (Mangiacotti et al. 2021), which were still available as frozen PBS solution. Following the same methodology described in (Mangiacotti et al. 2019b), we performed a novel Sodium dodecyl sulphate-polyacrylamide gel electrophoresis of the samples. From the freshly obtained gels, we carefully excised the bands occupying the molecular weight regions of interest, which were put in distinct tubes (one for each region) and analyzed with mass spectrometry (see supplementary material for protocol details). We performed protein identification using peptide-spectrum matching (Eng et al. 2011; Nesvizhskii 2014) against UniProt *Podarcis muralis* reference proteome (UP000472272; Bateman et al. 2021), with MS-GF + v2022.01.17 (Kim et al. 2008; Kim and Pevzner 2014) (see supplementary material for adopted settings and procedures). Protein identification was achieved when at least two different peptides match the same database entry with false detection rate below 0.01 (Mangiacotti et al. 2019).

## Results

### Protein–lipid covariation

**Fig. 1.**
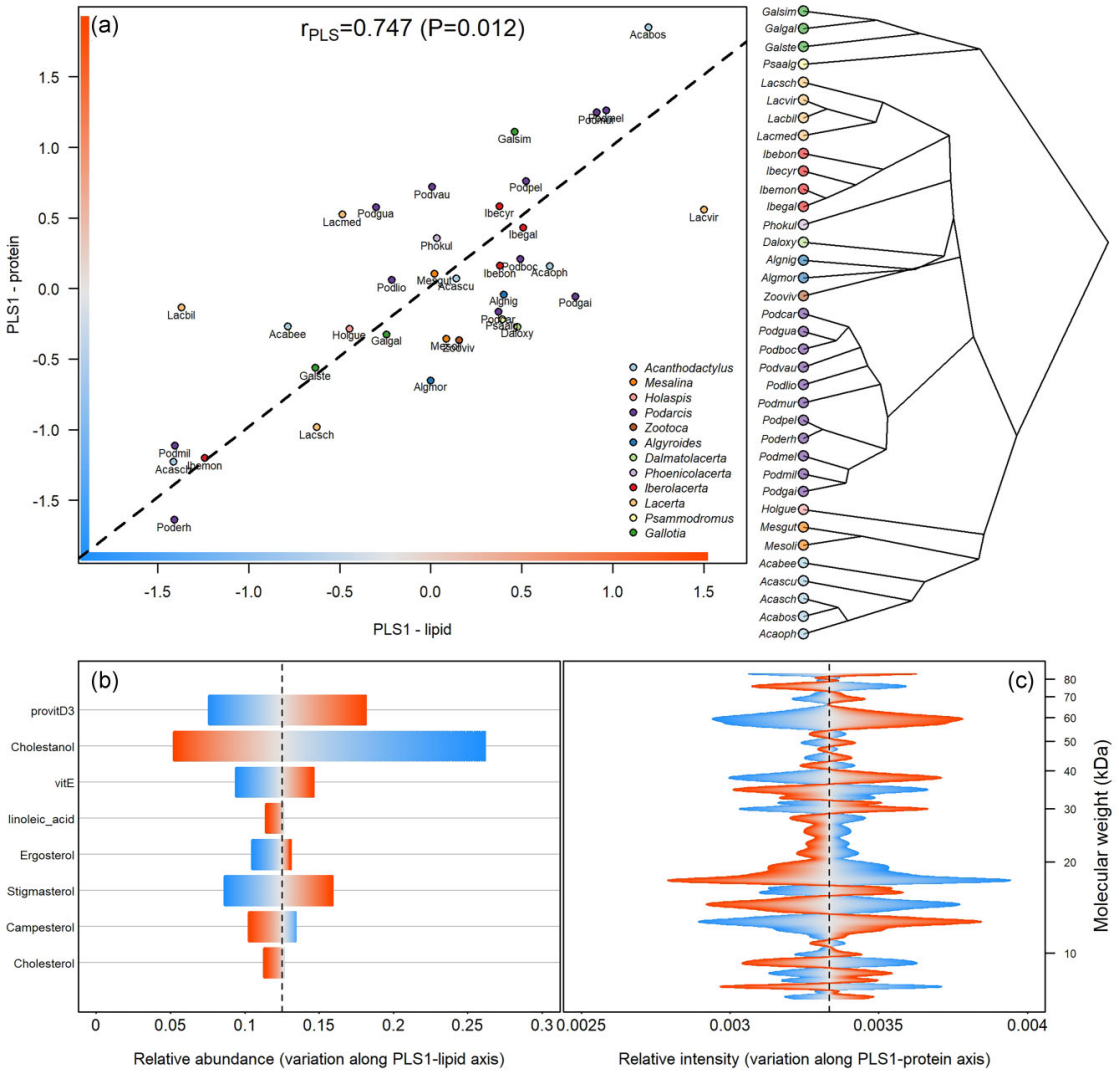
Representation of the phylogenetic partial least square regression between the compositions of the protein and lipid fraction of the femoral gland secretions of 36 lacertid species. (a) Plot of the scores along the first axis (PLS1) of the lipid- and the protein-matrix, respectively; only the main eight lipids were considered; the blue-to-red bars represent increasing scores along both axes; regression line is dashed; points are colored by genus. Right panel: cladogram of the species according to the phylogeny by (Garcia-Porta et al. 2019). (b) Relative abundance of the eight main lipids along the PLS1-lipid axis; color reflects score values as in panel A, in a way that light blue and orange correspond to low and high scores, respectively. (c) Relative intensity of the EPG along the molecular weight gradient associated with the PLS1-protein axis; light blue and orange coloration as in panels A and B.

**Fig. 2.**
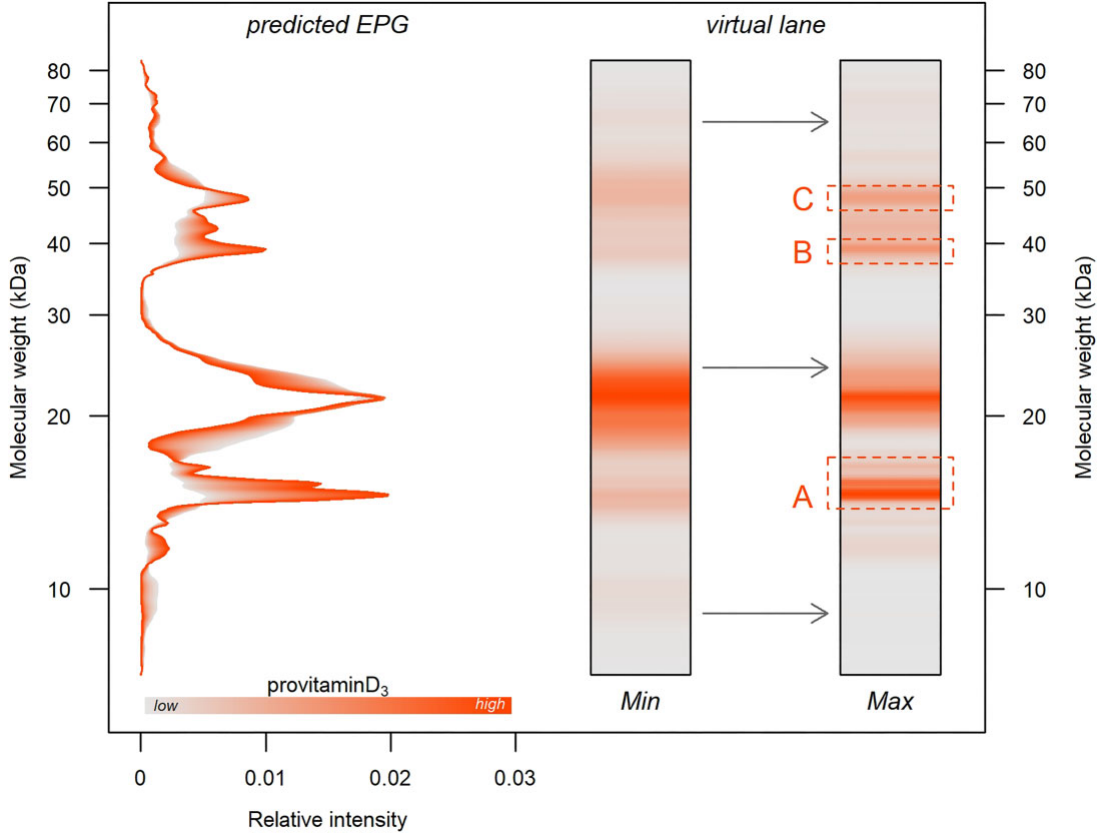
Effect of increasing abundance of provitamin D_3_ (grey-to-orange coloration) on the protein profile as predicted by the phylogenetic regression model. On the left, predicted electrophoretic profile (EPG); on the right, reconstruction of the electrophoretic running (virtual lane) corresponding to the minimum and maximum observed provitamin D_3_ abundances. Orange boxes highlight the three molecular weight regions (A, B, and C) of maximum variation, which were used to target mass spectrometry analysis (Table 2).

Phylogenetic partial least squares failed to identify a significant correlation between protein profiles (EPGs) and the class-level lipid composition (*r*_PLS_ = 0.625; *P* = 0.670; 9999 permutations), but revealed a significant association between the EPG pattern and the relative abundance of the eight lipids that are presumably active in chemical communication (*r*_PLS_ = 0.747; *P* = 0.012; 9999 permutations; Fig. 1A). Notably, protein variability seemed most related to the variation in the relative abundance of cholestanol, provitamin D_3_ (cholesta-5,7-dien-3-ol), stigmasterol, and vitamin E (α-tocopherol) according to their scores along the first axes (Fig. 1B). The most responsive regions of the protein profile (Fig. 1C) were roughly four: at 60 kDa, with a single peak involved; between 30 and 40 kDa, with three main peaks; between 12 and 20 kDa, with three recognizable peaks; and below 10 kDa, with two peaks. However, pGLS confirmed a significant main effect on EPG only for provitamin D_3_ (Table 1). As the abundance of this steroid lipid increased, the predicted electrophoretic pattern showed more intense and sharpen bands in three molecular weight regions (Fig. 2), respectively, at 47.7, 38.8, and between 14.5 and 16.2 kDa. The last two of these regions corresponded to two out of the four previously highlighted by partial least square analysis.

**Table 1.** ANOVA-like table from the multivariate phylogenetic regression model predicting protein composition (EPG) using the abundance of the eight main lipid compounds as independent variables. Type III sum of squares was used and significance of *F* values (pseudo-*F*) were tested using 9999 permutations (Adams and Collyer 2015). Bold denotes statistical significance (*P* < 0.05).

Variable	pseudo-*F*	*P*
Cholesterol	1.584	0.085
Campesterol	1.341	0.175
Stigmasterol	1.545	0.123
Ergosterol	1.177	0.288
Linoleic acid	1.450	0.122
Vitamin E	0.922	0.512
Cholestanol	1.161	0.280
**Provitamin D_3_**	**2.235**	**0.020**

**Fig. 3.**
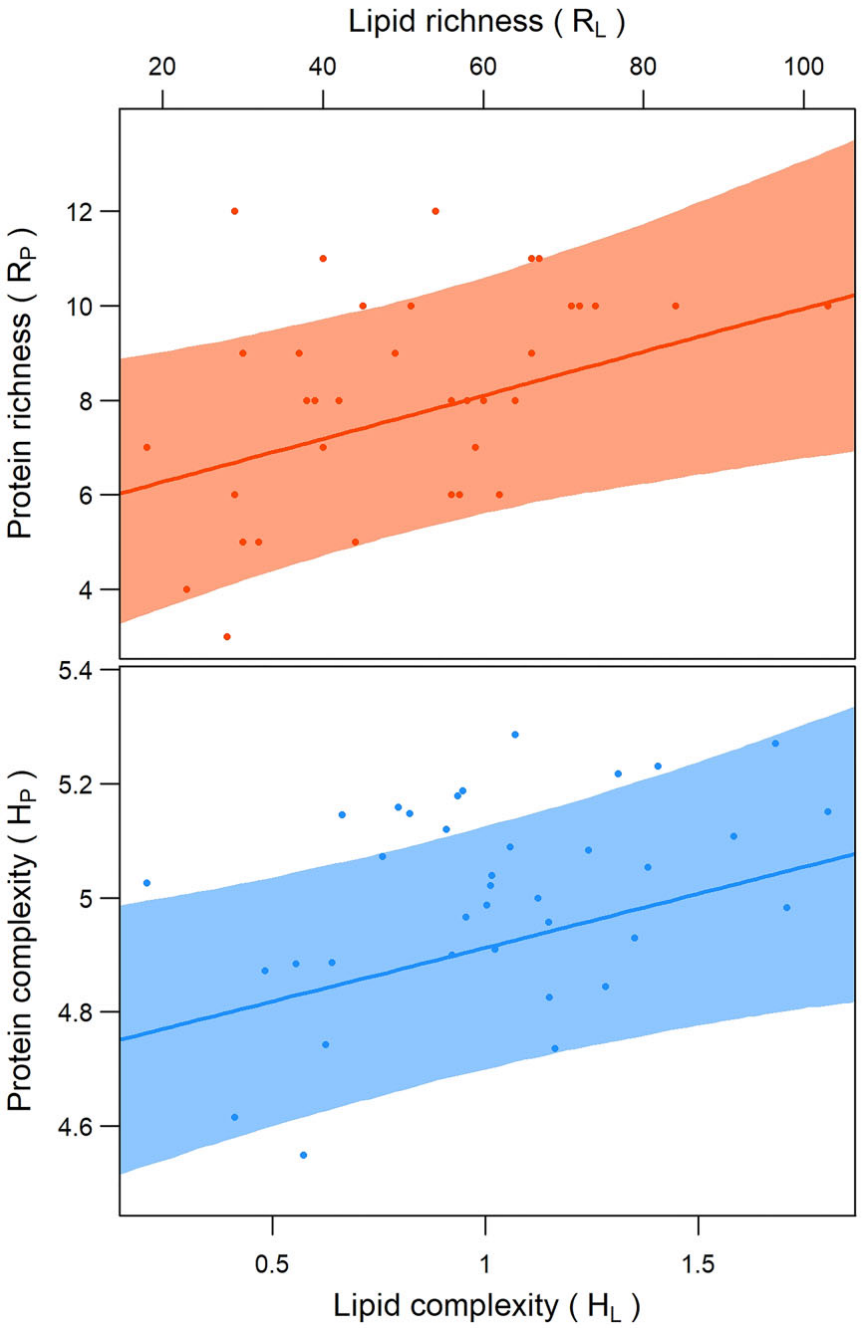
Relation between protein and lipid complexity according to the phylogenetic regression models. Upper panel: protein richness (R_P_), measured as the number of peaks along the EPG, on the *y*-axis, against lipid richness (*R*_L_ = no. of compounds), on the *x*-axis; solid line represents the average model prediction, grey area the 95% confidence interval of the line. Bottom panel: the same as the upper panel when using Shannon's entropy to measure protein (*H*_P_, *y*-axis) and lipid complexity (*H*_L_; *x*-axis).

Finally, the complexity of the protein pattern was found to be significantly correlated to lipid richness, whatever the chosen index were (*R*_P_ ∼ *R*_L_: pseudo-*F* = 6.040, *P* = 0.017; *H*_P_ ∼ *H*_L_: pseudo-*F* = 7.552, *P* = 0.010; 9999 permutations): as *R*_L_ increased, more peaks (i.e., protein clusters) were identified in the EPG (*R*_P_); the same occurred for the Shannon's indexes computed for protein- and lipid-profiles (Fig. 3).

### Protein identification

The electrophoretic run on *Podarcis muralis* samples allowed isolating the three molecular weight regions (bands at about 47.7, 38.8, and 14.5–16.2 kDa, respectively; Fig. S1) associated with the increased abundance in provitamin D_3_. Tandem mass spectrometry provided analyzable spectra, which led to peptide identifications in two out of the three gel bands. Specifically, five different peptides between 10 and 22 amino acids length were identified in the lighter band (A: 14.5–16.2 kDa; Table 2): three of them corresponded to the same database entry, namely “Protein disulfide-isomerase A6” (449 amino acids, 49.176 kDa), whose reported function is to catalyze the rearrangement of -S-S- bonds in proteins (Bateman et al. 2021). Despite the mismatch between predicted and observed molecular weight (about three times lower), the identification of multiple peptides from the same protein, each with low parental ion error and high score, makes the identification at protein level highly reliable. Two other peptides, not corresponding to the previous entry were also identified: a 12 amino acids sequence, showing low parental ion error and high score, and matching an uncharacterized protein (282 amino acids, 31.104 kDa; Table 2); a 22 amino acids length peptide, with high error and relatively low score, pointing to a large metallo-peptidase (1760 amino acids, 197.2 kDa; Table 2). The lack of multiple peptides matching the same protein, and the incompatible molecular weights (Table 2) suggested both identifications to be unlikely.

**Table 2.** Results of peptide–spectrum matching. Only target peptides with false detection rate < 0.01 were shown. Identifications are grouped by the three molecular weight regions considered (Band; Fig. 2 and S1), and by descending scoring. MW region = molecular weight region of the gel band according to the phylogenetic regression model; Sequence = amino acids sequence of the identified peptide; Length = length (number of amino acids) of the identified peptide; Error = difference between the measured and calculated parental ion mass (Da); Score = MSGF + spectrum *E*-value (−log_10_ transformed); FDR = false detection rate at the peptide level; Accession = uniprotKB accession id; Description = available uniprotKB protein description.

Band	MW region	Sequence	Length	Error	Score	FDR	Accession	Description
A	14.5–16.2	VGAVDADKHNSLGGQYGVR	19	0.013	24.861	0.000	A0A670J0 × 0	Protein disulfide-isomerase A6
		TSDAIVDAALSAIR	14	0.005	15.985	0.000		
		NRPEDYQGGR	10	0.005	9.465	0.000		
		LSIAVIHAECTK	12	0.000	14.990	0.000	A0A670IPJ5	Uncharacterized protein
		QSSHRTQWRFGSWTPCSTTCGK	22	1.003	8.066	0.000	A0A670J7U0	ADAM metallopeptidase with thrombospondin type 1 motif 9
B	38.8	YTQPDSCLGPLTLSGYEDR	19	0.016	22.735	0.000	A0A670JGA4	Carbonic anhydrase
		SFEAPFDGVWNFLASSLR	18	0.019	22.633	0.000		
		WCYDKPSCGPLTWR	14	0.009	20.115	0.000		
		GNSVPLNGEFSLSDLLRPGGR	21	0.016	18.868	0.000		
		MQDNYRPVQPLNNRR	15	0.003	18.426	0.000		
		ELLNKGDYPDVEMK	14	0.009	17.666	0.000		
		MQDNYRPVQPLNNR	14	0.007	17.569	0.000		
		SFEAPFDGVWNFLASSLRR	19	0.021	17.380	0.000		
		EAAKDPQGFVVLGFFIQK	18	0.012	16.105	0.000		
		KFTNSLFFTTK	11	0.006	14.234	0.000		
		FTNSLFFTTK	10	0.005	13.374	0.000		
		KQSPINIVTR	10	0.006	12.254	0.000		
		QSPINIVTR	9	0.005	10.991	0.000		
		VHPYVVKK	8	0.002	10.500	0.000		
		VHPYVVK	7	0.001	10.190	0.000		
		ITDKYYR	7	−0.001	9.810	0.000		
		HGGFGFPKNSGRLPGPR	17	0.964	8.666	0.000	A0A670HZ81	Uncharacterized protein
		KSTIWQFFSRLFSSSSSPPPAK	22	1.021	8.237	0.000	A0A670JMD3	Mitogen-activated protein kinase 8 interacting protein 3
		KEGDGNWKK	9	0.017	7.937	0.000	A0A670IGN5	Sodium/potassium-transporting ATPase subunit beta
		WFAKLADFRLFLPPR	15	−0.013	7.926	0.000	A0A670JQ55	Acid sensing ion channel subunit 2
C	47.7	*No match*						

Twenty different peptides were identified in the second band (B: 38.8 kDa, Table 2), ranging from 7 to 22 amino acids length. Eighteen corresponded to the same database entry, thus representing different fragments of the same protein, i.e., carbonic anhydrase (316 amino acids; 35.9 kDa; Table 2). The predicted molecular weight for this protein agreed with the position the band occupies in the SDS-PAGE according to the molecular weight ladder (Fig. S1), and it was not far from the weight region predicted by phylogenetic regression (Fig. 2). Together, these results made the identification highly credible. The remnant four sequences (Table 2) matched different entries, some of them showing high parental ion error, and all scoring worse than those corresponding to carbonic anhydrase. Again, the lack of multiple sequences matches, made these identifications poorly reliable.

In the end, no relevant match against the *P. muralis* proteome was found for the peptides obtained from the heavier band (C: 47.7 kDa; Table 2).

## Discussion

### Protein–lipid covariation

Empirical and theoretical work suggest that the proteinaceous and lipid fraction of lizard gland secretions may be intertwined, and thereby contribute (even with potentially different roles) to the functionality of lizard chemical display (Alberts 1990; Alberts and Werner 1993a; Mangiacotti et al. 2017, 2019a, 2020; Tellkamp et al. 2020; Ibáñez et al. 2022). Considering the Lacertid family, the quantitative phylogenetically-informed analysis confirm such a co-variation between proteins and lipids to occur. We indeed found a significant correlation between the proteinic and lipid composition when focussing on a subset of eight specific lipid compounds with known structural or signalling properties. Notably, EPGs clearly responded to the change in the relative proportion of cholestanol, provitamin D_3_, stigmasterol, and tocopherol, by increasing the concentration of proteins in certain molecular weight regions and decreasing it elsewhere. Additionally, an increase in provitamin D_3_ absolute concentration corresponded to a sharper expression of three EPG bands, suggesting the abundance of specific lipids to be related to that of specific proteins. Interestingly, a similar relation, involving almost the same molecular weight regions, was previously observed in the seasonal variation of the EPG of *P. muralis*, where the change in protein profiles matched that of the secreted provitamin D_3_ (Mangiacotti et al. 2019b). No relation was found when the composition of the lipid mixture is organized according to chemical classes (i.e., steroids, aldehydes, ketones, fatty acids, etc.). This may suggest that the protein–lipid association is not based on the occurrence in the lipid blend of specific chemical structure or functional groups, but rather of specific compounds, active in signal communication of pivotal to signal design (García-Roa et al. 2017b).

In the light of the support-to-lipid hypothesis, our finding of both general and specific correlations between the lipid and protein fraction in lizard secretions suggests additional, previously not theorized, roles for secretion proteins. Notably, proteins may contribute to enhance signal efficacy (*sensu*Endler 1993) by setting a “chemical environment” that inhibits more labile molecules (such as lipids) from oxidation, fast evaporation, or degradation (Alberts 1992, 1993; Wyatt 2010; Martín et al. 2016). In the iguanid *Amblyrhynchus cristatus* (Tellkamp et al. 2020) and the lacertid *Lacerta agilis* (Ibáñez et al. 2022), femoral gland secretions hold proteins with chemical properties that may serve in pH regulation (e.g., carbonic anhydrase), anti-oxidant function (e.g., thioredoxin), bacterial resistance (e.g., oncocin) or lipid-binding (e.g., fatty acid-binding protein 9), and not only in structural roles (e.g., keratin). This opens to the idea that proteins may be active in the mixture, and, by their quantitative and qualitative modulation, be involved in the very fine regulation of the efficacy, stability, and durability of the signal (Alberts 1992). Indeed, lizards typically deposit femoral secretions on bare substrates (Gabe and Saint Girons 1965; Cole 1966), and, consequently, secretions may experience hard and extreme conditions, ranging from dry to wet, over cold to hot, to high UV exposure and strong wind (Martín et al. 2016; Baeckens et al. 2018a; Campos et al. 2020). All above factors can heavily affect the ability of semiochemicals to withstand rapid degradation. Therefore, it would be worth a chemical blend enriched with compounds that counter the detrimental impact of climatic environment on the chemical properties of the blend itself (e.g., by regulating pH), or protecting signal molecules from them (e.g., from oxidation or biological degradation). A similar function has been already suggested for vitamin E (tocopherol), a lipophilic component of the secretions hypothesized to protect other semiochemicals against oxidation (López and Martín 2006; Gabirot et al. 2008). Contrary to the vitamin E case, where the function can be considered passive, i.e., protection comes from the reaction of tocopherol instead of other molecules, in the protein case, a higher-level interaction can be hypothesized, both because lipids may bond to proteins and because the latter may dynamically act on the chemical equilibrium of the mixture (Tellkamp et al. 2020; Ibáñez et al. 2022). These functional links may be expected to become gradually blurred by phylogenetic time (Garcia-Porta et al. 2019), which may explain why only one out of the eight target lipids (i.e., provitamin D_3_) was found to significantly predict EPG variation when explicitly tested in the regression model (Table 1; Fig. 2). Maybe, this relation may extend to more compounds if taxa sampling were at a lower phylogenetic level (e.g., within a single genus), where recent evolution and adaptation would be more dominant (Garcia-Porta et al. 2019).

Alternatively, or additionally, proteins may directly contribute to the semiochemical function (semiochemical hypothesis), being themselves part of the signal (Wyatt 2014). It has been shown that the proteinaceous fraction can convey information about class- or individual-identity (Alberts and Werner 1993b; Mangiacotti et al. 2019a, 2020), and it is potentially able to allow species level discrimination (Alberts 1991; Mangiacotti et al. 2019, 2021). Further, lizards’ vomeronasal system is able to discriminate proteins (Schwenk 1995; Cooper et al. 2002). Although no protein easily relatable to a communication function has been identified so far (Tellkamp et al. 2020; Ibáñez et al. 2022), this might reflect a bias of the database-driven protein identification process: Only the available annotated functions are associated with each identification, but multiple, unknown, roles cannot be excluded (Ibáñez et al. 2022). Even more so considering the peculiar chemo-physical properties of the secretions (i.e., dehydrated, lipophilic, and solid; Cole 1966; Mayerl et al. 2015), which are quite different from the physiological conditions where proteins usually exert their functions. If so, proteins might be part of a signature mixture (*sensu*Wyatt 2010) which, as a whole, may be used for individual recognition (Alberts 1992; Wyatt 2010; Mangiacotti et al. 2021), or may constitute single-component signals, as well (Hurst et al. 2001). The occurrence of a correlation between the two chemical components may support the former hypothesis, but targeted studies are needed to disentangle between these two alternatives.

As second support to the protein–lipid integration hypothesis comes from the positive correlation between the complexity of the two mixtures: As the number of lipid compounds raises, the structure of the protein electrophoretic patterns becomes more and more articulated, both considering the number of bands (i.e., identifiable protein clusters; *R*_P_), and their complexity (i.e., Shannon's Entropy; *H*_P_). The complexity of the lipid matrix responds to the environmental factors, increasing in mesic- compared to xeric-conditions (Baeckens et al. 2018a). It has been suggested that humidity may favor more complex signals due to the better performance of the chemoreceptive organs in humid conditions, which eventually promotes the recruitment of more compounds to increase redundancy or conveyable information (Baeckens et al. 2018a). In this scenario, proteins may do the same as lipids, both being a part of the signalling molecules, or providing the suitable chemical environment for lipids. In the latter case, moisture may require a wider range of proteins to contrast the faster degradation potential due to the wetter conditions (oxidation, pH modifications, and bio-degradation; Chamlin 1941; Bell 2020; Tapia et al. 2020). Although other causes may explain a so high variability of the lipophilic fraction (Baeckens et al. 2018a), the crucial point is the co-occurrence of the same pattern of variation in the lipophilic and proteinaceous counterparts, which strongly supports the hypothesis of a functional covariation between the two components, irrespective of the exact role of the proteins in the blend (semiochemical- or support-to-lipid hypothesis).

### Protein identification

Out of the three electrophoretic bands whose expression was related to the provitamin D_3_ abundance, two (A and B, Fig. 2 and S1) led to a reliable protein identification using tandem mass spectrometry. In both cases, a single protein is associated with each band, suggesting the actual number of proteins can be approximated by the number of bands in the gels (Alberts 1991; Mangiacotti et al. 2017).

The band A corresponds to protein disulfide-isomerase A6 (PDI), a 449 amino acid length protein known to catalyze the rearrangement of both intrachain and interchain disulfide bonds in proteins, and usually operating in the endoplasmic reticulum lumen (Ferrari and Söling 1999; Ali Khan and Mutus 2014). PDI has been already identified in femoral gland secretions of the sand lizard (Ibáñez et al. 2022) and marine iguana (Tellkamp et al. 2020), making our identification biologically relevant. Actually, the expected molecular weight (49.176 kDa; Bateman et al. 2021) is about three times the one estimated by the band position in the gel (between 14.4 and 16.0), but all the three identified peptides (which cover about 10% of the overall amino acid sequence) fall within the first thioredoxin domain of the protein (position: 21–138; Bateman et al. 2021), covering more than 36% of it. This let us hypothesize that the protein might not be integrally preserved in the secretion, but rather modified and fragmented: the estimated weight considering only the first domain ranges between 12.83 kDa (including only the 118 strictly pertaining amino acids) and 15.99 kDa (extending the sequence from the beginning to the second domain), which well matches the observed weight in the gel.

Concerning PDI functions in the mixture, notably related to provitamin D_3_, any possible hypotheses is subjected to the retaining its functional activity in a completely different environment compared to the usual ones (Ali Khan and Mutus 2014) and/or with only a part of its structure preserved. If so, different conjectures can be made: (1) it might stabilize other (undetected) proteins, linked to provitamin D_3_, by acting on their folding through the rearrangement of disulfide bonds (chaperone activity; Ali Khan and Mutus 2014); (2) it might control provitamin D_3_ oxidation through the thioredoxin-like domain (Gruber et al. 2006; Karala et al. 2009); and (3) it might be directly associated with provitamin D_3_, as a binding protein, since PDI can bind steroids (notably, 17β-estradiol; Fu et al. 2011).

The band B corresponds to further catalytic protein, i.e., carbonic anhydrase (CA), 316 amino acids length, 35.858 kDa weight (Bateman et al. 2021), whose function is to accelerate the carbon dioxide hydration and its reverse (i.e., bicarbonate dehydration; Tripp et al. 2001; Supuran 2016). CA has been detected in the femoral gland secretions of sand lizard (Ibáñez et al. 2022) and marine iguana, where it is among the most abundant proteins (Tellkamp et al. 2020). CA predicted weight agrees with the band position in the gel (Fig. S1), and the 22 identified peptides cover homogeneously 49% of the overall amino acid sequence, making its identification highly reliable. Assuming CA to be enzymatically active in the mixture, it is hard to specifically link its function to the provitamin D_3_ abundance. Besides a generic effect on pH regulation (Tellkamp et al. 2020; Ibáñez et al. 2022), indeed, CA might play an indirect role, by acting on the stability/activity of other compounds (proteins or lipids) which, in turn, may affect provitamin D_3_. For example, CA, by controlling pH, can regulate PDI catalytic activity (Wang and Narayan 2008).

Finally, for both CA and PDI, a semiochemical role cannot be excluded (see previous section of the discussion), in which case it should be expected to be related to that of provitamin D_3_.

## Conclusions

Our analysis revealed a relevant association between the lipid and a part of the protein fraction of the femoral gland secretions of lacertid lizards, which support the idea of an intertwined, more complex, system than previously thought. The exact functional relations between the two components are still far from being decrypted, but the occurrence of proteins with enzymatic behavior let us hypothesize that proteins may play an active role in the mixture, possibly conferring dynamic properties to the signal, i.e., making it able to somehow “react” to the predictable variation of the environmental conditions experienced by such cues. Of course, this hypothesis needs experimental supports from targeted studies: firstly, about the actual catalytic activity of the identified proteins in the secretions; secondly on the effect of such enzymatic functions on the blend properties. At the same time, we cannot exclude that proteins contribute to the semiochemical function of the blend, maybe forming with lipids a complex, individual-specific chemical profile (Wyatt 2010). Possibly, a correlative approach at a lower phylogenetic level may still be worthwhile, as it could help identifying other proteins and driving functional hypothesis formulation. As often the case, the availability of novel information leads to more questions, but our work provides new perspectives on the study of chemical communication mechanisms.

## Supplementary Material

obad016_Supplemental_File

## Data Availability

The data underlying this article are available in Zenodo repository, doi: 10.5281/zenodo.7036727.
