# Supplementary material for: Protein–lipid Association in Lizard Chemical Signals
Source: Integr Org Biol. 2023 May 8;5(1):obad016. doi: 10.1093/iob/obad016 (PMC10205002; doi:10.1093/iob/obad016)
Supplement: obad016_Supplemental_File [file obad016_supplemental_file.pdf]

M1. Note on samples collection

Although the specifics on the sampling collection have been reported in earlier studies (García-Roa et al. 2017; Baeckens et al. 2018a), we briefly summarise here the basic field protocol. Sampling was carried out between 2002 and 2016 (Table S1), during seasonal periods where glandular activity reaches its maximum (i.e., the breeding season; Cole 1966; Alberts et al. 1992; Mangiacotti, Pezzi, et al. 2019). Lizards were captured by hand or noose, and femoral glands secretions were obtained by gently pressing along the thigh with the help of a steel spatula, a non-invasive technique which causes minimal stress to the animals. No lizards were killed or injured during the study. Once transferred into individual vials, the secretions were kept at -20 °C until analysis. All lizards were released, healthy, at their capture point. The experimental design and procedure complied the ARRIVE guidelines (https://arriveguidelines.org/), and was in accordance with University of Antwerp (Belgium) animal welfare standards and protocols (ECD 2014-32). Permits or licences for each state are listed from (Baeckens et al. 2018a): Croatia (UP/I-612-07/14-48/111 & UP/I-612-07/14-48/33), The Netherlands (FF/74A/2015/009), Israel (2014/40323), SA Free State Province (S54C-515022511060), SA Eastern Cape Province (CRO 45/15CR & 46/15CR), SA Western Cape Province (0056-AAA041-00093), SA Northern Cape Province (FAUNA 229/2015 & 230/2015); SA Limpopo Province (0092-MKT001-00004); Spain (Captures of lizards and sampling procedures were performed under different licenses for the Environmental Agencies of the different Regional Governments of Spain where lizards were studied); Greece (all Greek species were collected in accordance with the Hellenic National Legislation, Presidential Decree 67/81).

M2. Mass Spectrometry and protein identification protocol

Sample preparation. From the Sodium dodecyl sulphate-polyacrylamide gel (Fig. S1), we carefully excised the bands occupying the molecular weight regions of interest, and put them in distinct tubes (one for each region). We prepared the samples for mass spectrometry analysis as follows: (1) we completely de-stained the gel pieces by washing twice with 200 μL of 100 mM ammonium bicarbonate buffer pH 7.8, 50 % acetonitrile (ACN) and keeping under stirring overnight; (2) we dehydrated the samples with 100 μL ACN; (3) we performed reduction of the samples by adding 100 μL of 10 mM Dithiothreitol (DTT) solution (30 min at 37°C), replaced with 100 μL of 55 mM iodoacetamide for 45 min at 60°C; (4) we washed the gel pieces twice with 200 μL of 100 mM ammonium bicarbonate for 10 min; (5) we dehydrated by addition of 100 μL of ACN; (6) we removed ACN and dried samples under vacuum; (7) we performed protein digestion overnight at 37°C by adding 100 μL of 100 mM ammonium bicarbonate buffer pH 7.8, containing 20 ng/μL sequencing grade trypsin (Promega, Madison, WI, USA); (8) we extracted the obtained peptides by adding twice 100 μL of 50 % ACN in water, 5 % formic acid (FA), and pooling the supernatant from each step after stirring and centrifuging; (9) we dried each pool and stored at -20°C until mass spectrometry analysis.

Mass spectrometry. Before analysis, peptide mixtures were first solubilized in 100μL of 0.1% formic acid (FA). All analyses were carried out with a LC unit (ExionLCTM AD) equipped with a column oven thermostated at 40°C, an autosampler cooled at 10°C and a binary gradient pump system. MS instrument consists of a high resolution QTOF mass spectrometer (AB Sciex X500B) equipped with a Turbo V Ion source and a Twin Sprayer ESI (electrospray ionization) probe, controlled by SCIEX OS 2.1 software. Peptides were separated by reverse phase (RP) HPLC on a Hypersil Gold (Thermo Fisher Scientific, USA) C18 column (150 × 2.1 mm, 3 μm particle size, 175 Å pore size) using a linear gradient (2-50 % solvent B in 15 min) in which solvent A consisted of 0.1 % aqueous FA and solvent B of acetonitrile (ACN) containing 0.1 % FA. Flow rate was 0.2 mL/min. Mass spectra were generated in positive polarity under constant instrumental conditions: ion spray voltage 4500 V, declustering potential 100 V, curtain gas 30 psi, ion source gas 1 40 psi, ion source gas 2 45 psi, temperature 350 °C, collision energy 10 V. Spectra were acquired with SCIEX OS 2.1 software.

Database searching. We used MS-GF+ v2022.01.17 (Kim et al. 2008; Kim and Pevzner 2014) for protein identification by peptide-spectrum matching (Eng et al. 2011; Nesvizhskii 2014). We set the algorithm as follows (Tellkamp et al. 2020): tolerance, 30 ppm; charge range, 1 – 6+; range of peptide length, 7 – 70; isotope error 0 – 1 Da; cleavage, tryptic; post translational modification, fix carbamidomethylation of cysteine (Creasy and Cottrell 2004; Eng et al. 2011; Schittmayer et al. 2016). Searching was performed against UniProt *Podarcis muralis* reference proteome (UP000472272; Bateman et al. 2021), consisting in 36,445 protein sequences obtained from the *P. muralis* genome (Andrade et al. 2019), to which pig trypsin and human keratin sequences (retrieved from uniprot.org) were added to account for contamination. To maximize identification power, a two-stages, target-decoy approach was run (Craig and Beavis 2003; Jeong et al. 2012; Mangiacotti, Fumagalli, et al. 2019). An unbiased false detection rate (FDR) was applied at peptide level (Everett et al. 2010; Bern and Kil 2011), using spectrum E-value as the reference score (Jeong et al. 2012), and peptides with FDR > 0.01 were filtered out from the final list. Protein identification was achieved when at least two different peptides match the same database entry (Mangiacotti, Fumagalli, et al. 2019). Call to MSGF+ and all post-processing analyses were done in R v3.5.2 (R Core Team 2018), using the packages `mzID` (Pedersen et al. 2016), `Biostrings` (Pagès et al. 2017), `stringr` (Wickham 2018).

Species	Latitude	Longitude	N	Month	Year	R_P	H_P	R_L	H_L
Acanthodactylus beershebensis	31.07	34.84	4	April	2014	8	4.91	56	1.02
Acanthodactylus boskianus	30.95	34.59	7	April	2014	8	5.19	38	0.95
Acanthodactylus opheodurus	29.94	35.08	4	June	2014	12	5.18	29	0.93
Acanthodactylus schreiberi	32.00	34.79	5	April	2014	9	5.22	37	1.31
Acanthodactylus scutellatus	32.00	34.79	5	April	2014	8	4.96	39	1.15
Algyroides moreoticus	37.25	21.01	4	May	2014	9	5.15	30	0.66
Algyroides nigropunctatus	45.10	14.34	5	May	2013	11	4.99	67	1.00
Dalmatolacerta oxycephala	44.69	14.39	11	May	2013	6	5.03	29	0.20
Gallotia galloti	28.08	-16.62	9	April	2013	10	5.05	103	1.38
Gallotia simonyi	27.76	-17.99	22	June	2014	6	4.93	57	1.35
Gallotia stehlini	27.74	-15.60	5	April	2013	5	4.74	32	0.62
Holaspis guentheri	-3.81	37.63	17	August	2014	10	5.08	45	1.24
Iberolacerta bonnali	42.61	0.18	7	June	2013	8	5.07	42	0.76
Iberolacerta cyreni	40.79	-4.01	15	May	2003	5	4.55	44	0.57
Iberolacerta galani	42.41	-6.41	10	May	2010	9	4.97	66	0.95
Iberolacerta monticola	40.32	-7.60	19	May	2006	9	4.87	49	0.48
Lacerta bilineata	42.73	-3.41	7	May	2013	8	5.02	42	1.01
Lacerta media	33.21	35.77	6	April	2014	10	5.12	71	0.91
Lacerta schreiberi	40.78	-4.07	15	April	2005	10	5.09	51	1.06
Lacerta viridis	47.35	19.76	22	April	2008	7	5.00	40	1.12
Mesalina guttulata	30.83	34.75	5	April	2014	4	4.85	23	1.28
Mesalina olivieri	31.04	35.07	6	April	2014	3	4.83	28	1.15
Phoenicolacerta kulzeri	33.30	35.79	5	April	2014	5	4.88	30	0.55
Podarcis bocagei	42.74	-9.08	9	May	2007	6	5.15	56	0.82
Podarcis carbonelli	40.50	-6.09	6	May	2007	8	5.16	60	0.79
Podarcis erhardii	37.83	24.85	7	May	2014	11	5.04	40	1.02
Podarcis gaigeae	38.95	24.52	6	May	2014	10	5.15	72	1.80
Podarcis guadarramae	40.73	-4.04	21	April	2002	6	4.89	62	0.64
Podarcis liolepis	42.75	1.84	12	May	2012	7	4.90	59	0.92
Podarcis melisellensis	45.10	14.34	21	May	2013	12	5.29	54	1.07
Podarcis milensis	36.69	24.44	7	May	2014	10	5.11	74	1.58
Podarcis muralis	45.24	9.23	23	May	2011	8	4.98	64	1.71
Podarcis peloponnesiacus	37.24	22.46	5	May	2014	11	5.27	66	1.68
Podarcis vaucheri	35.18	-2.43	8	April	2012	10	5.23	84	1.40
Psammodromus algirus	40.73	-4.37	22	May	2004	8	4.74	58	1.16
Zootoca vivipara	44.41	3.74	35	April	2005	7	4.62	18	0.41

Table S1. List of the species included in the study ordered by their scientific name with information about the sampling locality, size (N), and period (month and year), and the chemical complexity of proteinaceous (R_P and H_P) and lipid (R_L and H_L) fractions of the femoral gland secretions: R_P = number of bands in the electrophoretic profile; H_P = Shannon entropy of the electrophoretic profile; R_L = total number of distinct lipid compounds; H_L = Shannon entropy of the lipid profile. Sampling data and R_L were taken from Baeckens et al. (2018b), R_P, H_P, and H_L were computed starting from available data. See methods for details.

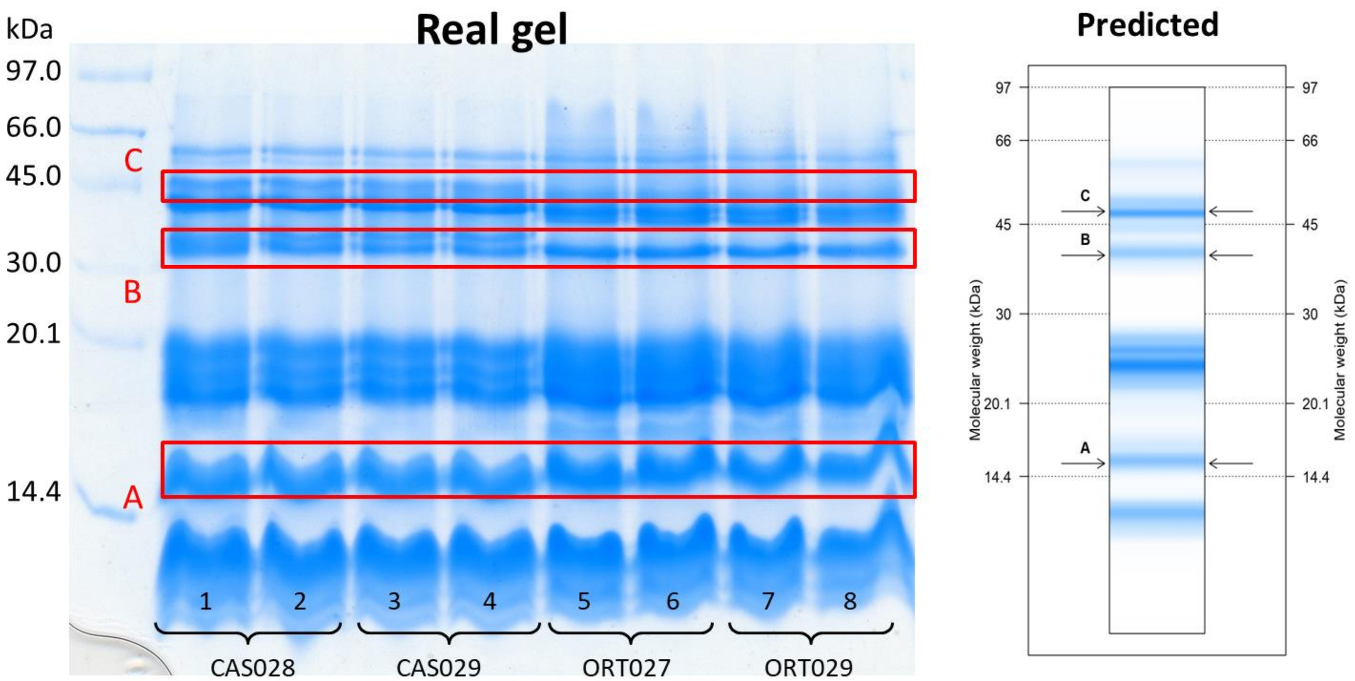

Figure S1. *On the left.* SDS-PAGE gel of the *Podarcis muralis* samples used to retrieve the bands correlated with pro-vitamin D_3 abundance as predicted by pGLS models. The gel pieces from the same molecular weight region were pooled before mass spectrometry analysis. Ladder lanes are marked with the corresponding molecular weights. We duplicated each of the four samples (ID code at the bottom of right panel) in two adjacent lanes to increase protein concentration in each region. *On the right*. Virtual lane reconstruction of the *Podarcis muralis* gel with arrows and letters indicating the predicted position of the bands responding to increase pro-vitamin D_3; it was used to drive bands identification in the real gel.

REFERENCES

Alberts AC, Pratt NC, Phillips JA. 1992. Seasonal productivity of lizard femoral glands: Relationship to social dominance and androgen levels. Physiology & Behavior 51:729–33.

Andrade P, Pinho C, Pérez i de Lanuza G, Afonso S, Brejcha J, Rubin C-J, Wallerman O, Pereira P, Sabatino SJ, Bellati A, Pellitteri-Rosa D, Bosakova Z, Bunikis I, Carretero MA, Feiner N, Marsik P, Paupério F, Salvi D, Soler L, While GM, Uller T, Font E, Andersson L, Carneiro M. 2019. Regulatory changes in pterin and carotenoid genes underlie balanced color polymorphisms in the wall lizard. Proceedings of the National Academy of Sciences 116:5633–42.

Baeckens S, Martín J, García-Roa R, Pafilis P, Huyghe K, Van Damme R. 2018a. Environmental conditions shape the chemical signal design of lizards. Functional Ecology 32:566–80.

Baeckens S, Martín J, García-Roa R, Pafilis P, Huyghe K, Van Damme R. 2018b. Environmental conditions shape the chemical signal design of lizards. Functional Ecology 32:566–80.

Bateman A, Martin MJ, Orchard S, Magrane M, Agivetova R, Ahmad S, Alpi E, Bowler-Barnett EH, Britto R, Bursteinas B, Bye-A-Jee H, Coetzee R, Cukura A, Silva A Da, Denny P, Dogan T, Ebenezer TG, Fan J, Castro LG, Garmiri P, Georghiou G, Gonzales L, Hatton-Ellis E, Hussein A, Ignatchenko A, Insana G, Ishtiaq R, Jokinen P, Joshi V, Jyothi D, Lock A, Lopez R, Luciani A, Luo J, Lussi Y, MacDougall A, Madeira F, Mahmoudy M, Menchi M, Mishra A, Moulang K, Nightingale A, Oliveira CS, Pundir S, Qi G, Raj S, Rice D, Lopez MR, Saidi R, Sampson J, Sawford T, Speretta E, Turner E, Tyagi N, Vasudev P, Volynkin V, Warner K, Watkins X, Zaru R, Zellner H, Bridge A, Poux S, Redaschi N, Aimo L, Argoud-Puy G, Auchincloss A, Axelsen K, Bansal P, Baratin D, Blatter MC, Bolleman J, Boutet E, Breuza L, Casals-Casas C, de Castro E, Echioukh KC, Coudert E, Cuche B, Doche M, Dornevil D, Estreicher A, Famiglietti ML, Feuermann M, Gasteiger E, Gehant S, Gerritsen V, Gos A, Gruaz-Gumowski N, Hinz U, Hulo C, Hyka-Nouspikel N, Jungo F, Keller G, Kerhornou A, Lara V, Le Mercier P, Lieberherr D, Lombardot T, Martin X, Masson P, Morgat A, Neto TB, Paesano S, Pedruzzi I, Pilbout S, Pourcel L, Pozzato M, Pruess M, Rivoire C, Sigrist C, Sonesson K, Stutz A, Sundaram S, Tognolli M, Verbregue L, Wu CH, Arighi CN, Arminski L, Chen C, Chen Y, Garavelli JS, Huang H, Laiho K, McGarvey P, Natale DA, Ross K, Vinayaka CR, Wang Q, Wang Y, Yeh LS, Zhang J. 2021. UniProt: The universal protein knowledgebase in 2021. Nucleic Acids Research 49:D480–89.

Bern M, Kil YJ. 2011. Comment on “unbiased statistical analysis for multi-stage proteomic search strategies.” Journal of Proteome Research 10:2123–27.

Cole CJ. 1966. Femoral glands in lizards: a review. Herpetologica 22:199–206.

Craig R, Beavis RC. 2003. A method for reducing the time required to match protein sequences with tandem mass spectra. Rapid Communications in Mass Spectrometry 17:2310–16.

Creasy DM, Cottrell JS. 2004. Unimod: Protein modifications for mass spectrometry. Proteomics 4:1534–36.

Eng JK, Searle BC, Clauser KR, Tabb DL. 2011. A Face in the Crowd: Recognizing Peptides Through Database Search. Molecular & Cellular Proteomics 10:R111.009522.

Everett LJ, Bierl C, Master SR. 2010. Unbiased Statistical Analysis for Multi-Stage Proteomic Search Strategies. Journal of Proteome Research 9:700–707.

García-Roa R, Jara M, López P, Martín J, Pincheira-Donoso D. 2017. Heterogeneous tempo and mode of evolutionary diversification of compounds in lizard chemical signals. Ecology and Evolution 7:1286–96.

Jeong K, Kim S, Bandeira N. 2012. False discovery rates in spectral identification. BMC bioinformatics 13 Suppl 1:S2–S2.

Kim S, Gupta N, Pevzner PA. 2008. Spectral probabilities and generating functions of tandem mass spectra: A strike against decoy databases. Journal of Proteome Research 7:3354–63.

Kim S, Pevzner PA. 2014. MS-GF+ makes progress towards a universal database search tool for proteomics. Nature Communications 5:5277.

Mangiacotti M, Fumagalli M, Cagnone M, Viglio S, Bardoni AM, Scali S, Sacchi R. 2019. Morph-specific protein patterns in the femoral gland secretions of a colour polymorphic lizard. Scientific Reports 9:8412.

Mangiacotti M, Pezzi S, Fumagalli M, Coladonato AJ, d’Ettorre P, Leroy C, Bonnet X, Zuffi MAL, Scali S, Sacchi R. 2019. Seasonal Variations in Femoral Gland Secretions Reveals some Unexpected Correlations Between Protein and Lipid Components in a Lacertid Lizard. Journal of Chemical Ecology 45:673–83.

Nesvizhskii AI. 2014. Proteogenomics: concepts, applications and computational strategies. Nat Methods 11:1114–25.

Pagès H, Aboyoun P, Gentleman R, DebRoy S. 2017. Biostrings: Efficient manipulation of biological strings. R package version 2460.

Pedersen TL, with contributions from Laurent Gatto VAP, Gibb. S. 2016. mzID: An mzIdentML parser for R. .

R Core Team. 2018. R: a language and environment for statistical computing. .

Schittmayer M, Fritz K, Liesinger L, Griss J, Birner-Gruenberger R. 2016. Cleaning out the Litterbox of Proteomic Scientists Favorite Pet: Optimized Data Analysis Avoiding Trypsin Artifacts. Journal of Proteome Research 15:1222–29.

Tellkamp F, Lang F, Ibáñez A, Abraham L, Quezada G, Günther S, Looso M, Tann FJ, Müller D, Cemic F, Hemberger J, Steinfartz S, Krüger M. 2020. Proteomics of Galápagos Marine Iguanas Links Function of Femoral Gland Proteins to the Immune System. Molecular and Cellular Proteomics 19:1523–32.

Wickham H. 2018. stringr: Simple, Consistent Wrappers for Common String Operations. .
